# Opportunities and challenges to implementing mRNA-based vaccines and medicines: lessons from COVID-19

**DOI:** 10.3389/fpubh.2024.1429265

**Published:** 2024-08-08

**Authors:** Shehzad M. Iqbal, Andrew M. Rosen, Darin Edwards, Ana Bolio, Heidi J. Larson, Mariana Servin, Marcy Rudowitz, Andrea Carfi, Francesca Ceddia

**Affiliations:** ^1^Moderna, Inc., Cambridge, MA, United States; ^2^London School of Hygiene and Tropical Medicine, University of London, London, United Kingdom; ^3^Institute for Health Metrics and Evaluation, University of Washington, Seattle, WA, United States

**Keywords:** mRNA vaccines and therapeutics, COVID-19, mRNA vaccine development, public trust, vaccine hesitancy, vaccine confidence

## Abstract

The messenger RNA (mRNA) platform emerged at the forefront of vaccine development during the COVID-19 pandemic, with two mRNA COVID-19 vaccines being among the first authorized globally. These vaccines were developed rapidly. Informed by decades of laboratory research, and proved to be safe and efficacious tools for mitigating the global impact of the COVID-19 pandemic. The mRNA platform holds promise for a broader medical application beyond COVID-19. Herein, we provide an overview of this platform and describe lessons learned from the COVID-19 pandemic to help formulate strategies toward enhancing uptake of future mRNA-based interventions. We identify several strategies as vital for acceptance of an expanding array of mRNA-based vaccines and therapeutics, including education, accurate and transparent information sharing, targeted engagement campaigns, continued investment in vaccine safety surveillance, inclusion of diverse participant pools in clinical trials, and addressing deep-rooted inequalities in access to healthcare. We present findings from the Global Listening Project (GLP) initiative, which draws on quantitative and qualitative approaches to capture perceptions and experiences during the COVID-19 pandemic to help design concrete action plans for improving societal preparedness for future emergencies. The GLP survey (>70,000 respondents in 70 countries) revealed tremendous disparities across countries and sociodemographic groups regarding willingness to accept novel mRNA vaccines and medicines. The comfort in innovations in mRNA medicines was generally low (35%) and was marginally lower among women (33%). The GLP survey and lessons learnt from the COVID-19 pandemic provide actionable insights into designing effective strategies to enhance uptake of future mRNA-based medicines.

## 1 Introduction

Tailored healthcare campaigns that engage the public and provide resources to address specific health needs are integral to enhancing health and preventing disease (1). The success of vaccination campaigns is predicated on a multitude of factors, including public trust in health authorities and political leadership, access to vaccines, and perceptions of vaccination (2, 3). These factors vary across countries (2, 4–8) and intersect with more dynamic influences (e.g., rapidly evolving policy recommendations, media coverage) (2, 3, 8, 9). During the COVID-19 pandemic, the relationship between vaccine hesitancy, sociodemographic characteristics, and political leaning became notable (10–12). Social inequalities were associated with disparate access to care and differential health burden (11, 13), influencing vaccine perceptions and potentially shaping future vaccine behavior. Quantitative measures and benchmarking can provide actionable insights on societal preparedness to mitigate the long-term impact of healthcare crises, e.g., by addressing the gaps between public perceptions and evidence-based information, and targeting trust-building interventions to appropriate demographic groups (14).

Messenger RNA-based vaccines (hereafter mRNA vaccines) were among the primary authorized vaccines against SARS-CoV-2 during the COVID-19 pandemic (15). Although mRNA research has been ongoing for several decades (16), the use of the mRNA platform for vaccines came into the limelight only during the COVID-19 pandemic (15, 17). The novelty of this mode of producing vaccines generated concerns in the public regarding perceived lack of adequate testing of side-effects of mRNA vaccines (2). Herein, we provide an overview of how the mRNA platform works and discuss how lessons learned from the pandemic can inform strategies to enhance trust and facilitate uptake of mRNA-based vaccines and therapeutics beyond COVID-19. We present novel data from a Global Listening Project (GLP) survey (18) showcasing nationwide diversity in the pandemic-era experiences of mRNA-based vaccines and medicines. These data reveal a multifactorial basis underlying acceptance of mRNA-based medicines, highlighting the need for improved communication on this topic and equitable access to care in the time of crisis.

## 2 The mRNA vaccine platform

Messenger RNA is an essential molecule involved in relaying genetic information encoded in DNA to the production of proteins (19–21). Vaccines based on mRNA can be designed to selectively produce key proteins from pathogens that stimulate a specific immune response, thereby protecting from illness (16, 22, 23). mRNA contains a transcript that directs the production of highly immunogenic proteins by the cells that take up the vaccine and stimulate the immune system the same way as a natural infection (16, 24–26). The protein encoded by mRNA represents one component of the pathogen, and therefore is unable to cause disease (20).

The constituents of mRNA vaccines are synthetic, non-replicating mRNA molecules that approximate the size and composition of naturally occurring mRNA (15, 26), encapsulated by lipid nanoparticles (LNPs) that serve to protect the mRNA from degradation and enable targeted cellular delivery (19, 24, 27, 28). Following administration, the mRNA is rapidly degraded by normal physiological processes (20, 29–31), while the naturally occurring lipids in the LNP vehicles are assumed to be biologically degraded similar to their endogenous analogs (27, 31). The synthetic amino lipid constituent of LNP is rapidly cleared from blood in rodent models (27, 31). Notably, mRNAs do not enter the cell nucleus and therefore cannot integrate into the cellular genome (16).

The key advantages of mRNA over other vaccine platforms (Table 1) are the precision in protein design, the flexibility to reconfigure protein formulations toward enhancing immunogenicity or developing combination vaccines to target multiple pathogens, and the speed at which vaccines can be manufactured and updated (e.g., allowing expeditious updates to target evolving or emerging strains) (24, 28). The manufacturing of mRNA involves standardized chemical processes with reagents that can be rapidly repurposed independently of the encoded protein (28) without the need for adjuvants (26). The specificity and flexibility of the platform allow for iterative improvements in protein design and make the mRNA approach intrinsically faster and scalable up to hundreds of millions of doses (28, 32).

**Table 1:** Summary of key differences between the mRNA platform and other vaccine technologies.

Vaccine type		Advantages	Disadvantages
*Live-attenuated* A weakened or non-infectious pathogen (82, 83)	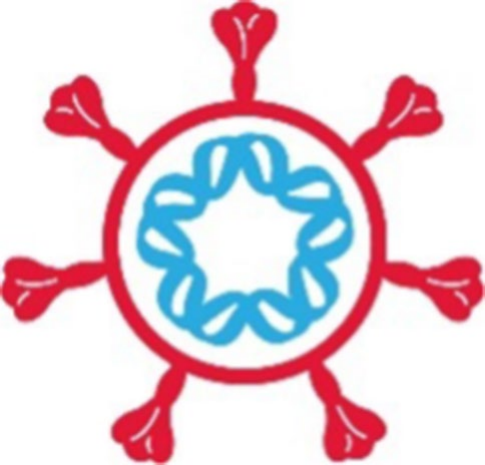	Mimics natural infection (83) Simple design (82) Robust immunogenicity (82) Long lasting (82)	Stringent biocontainment (82) Cold transport requirements (82) Strong adverse immune reactions in vulnerable populations (82) Prone to reverse mutations to an infectious strain (82)
*Inactivated* A fully killed pathogen (82, 83)	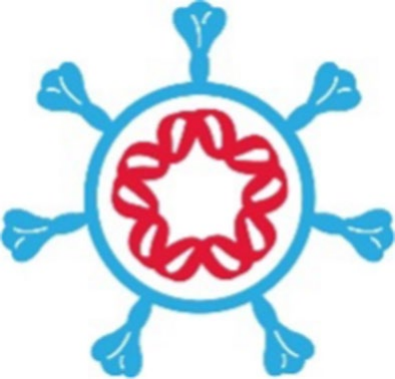	Broader immune response (82) Safer than live-attenuated (83) Stable and scalable (83)	Potential epitope alteration (83) Typically requires booster doses (82)
*Subunit* Purified or recombinant protein/peptide of the target pathogen (82–84)	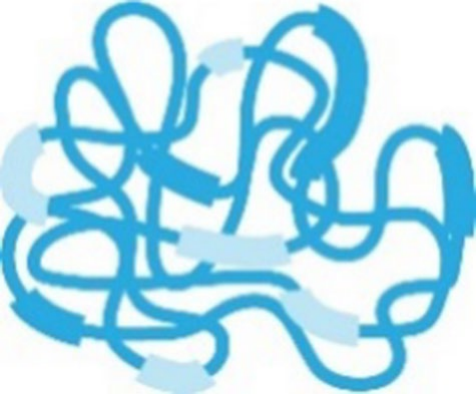	Contain no live components (83) Favorable safety profile (83) Flexible, enabling combination vaccines (83) Stable and scalable (83)	Low immunogenicity, often requiring an adjuvant or a conjugate (83) Frequent boosting required (83) Labor-and time-intensive to manufacture (83)
*mRNA* Nucleic acid vaccine (83, 84)	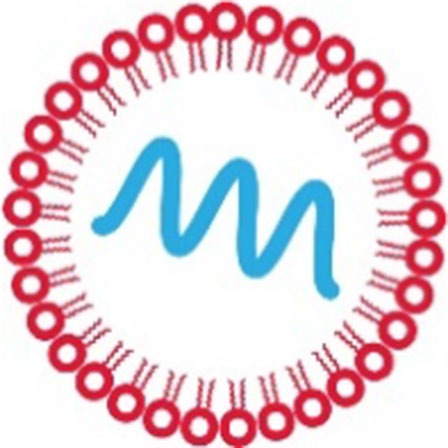	Precise protein design (28) Modifiable to target new pathogens (28) No risk of insertional mutagenesis (80) Low risk of toxicity (28, 80) Rapid inexpensive production (28, 80) Well-tolerated and effective (28)	Low temperature required for storage and transportation (83)
*DNA* DNA sequence (82)	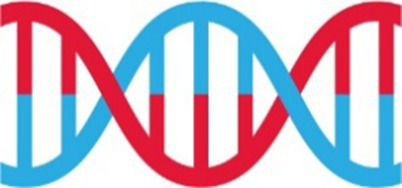	Adaptable to target new pathogens (83, 85) Thermostability at refrigerated and ambient temperatures (85)	Complexity of delivery and increased cost due to a requirement for a device to enhance cellular uptake (85) Lower antibody responses compared to mRNA and adenoviral vaccines (85) Risk of genomic integration (82)
*Viral vectored* Recombinant protein of the target pathogen in the carrier virus vector (84)	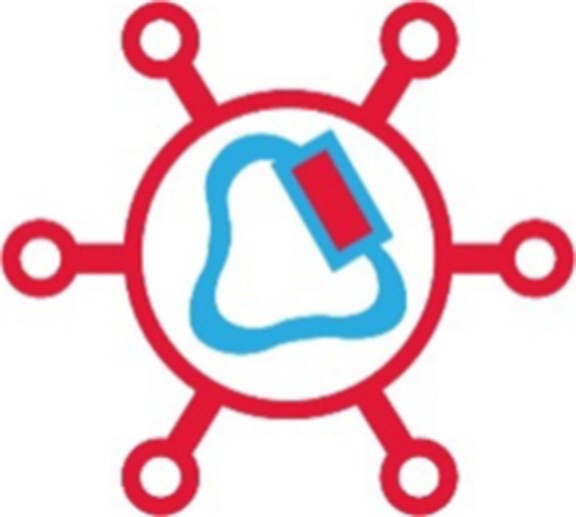	Flexible; can target multiple pathogens (28) Rapid manufacturing and scale-up (28) Potent and stable, supporting single-shot administration (28) Cost-effective (82) Thermostability at refrigerated temperatures (82)	Response dampened by pre-existing immunity against vector (83) Risk of genomic integration (83) Rare adverse events of thrombosis and thrombocytopenia associated with COVID-19 vaccines (86)
*Toxoid* Inactivated toxin of a disease-causing agent (82, 83)	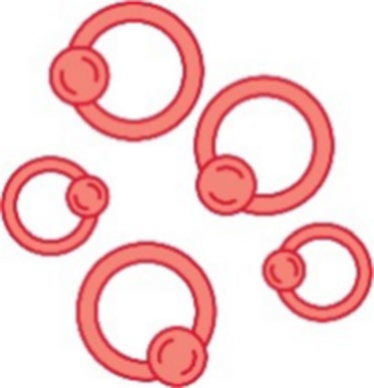	Non-virulent (83) Stable; long-lasting storage (83)	Local injection-site reactions (83) Immune responses may not be robust enough, necessitating booster doses (83)

Since the discovery of mRNA in 1961, its medical application has been hampered by various factors, including short half-life and inflammatory properties (28, 33, 34). A breakthrough discovery in 2005 showing that replacing uridine with pseudouridine decreased the degree of mRNA-driven inflammation (28, 34, 35), and additional technological advancements in encapsulating mRNA in LNPs were the key milestones underlying the development of mRNA vaccines (16, 31). With the declaration of the COVID-19 pandemic, two mRNA COVID-19 vaccines, mRNA-1273 (Spikevax, Moderna, Inc., Cambridge, MA, United States) and BNT162b2 (Comirnaty; Pfizer, Inc. New York, NY, United States) were among the first vaccines against SARS-CoV-2 authorized for emergency use worldwide (15). These approvals were based on the data from pivotal Phase 3 randomized clinical trials involving >30,000 participants, which demonstrated high efficacy (>90%) and a favorable risk–benefit profile (36, 37). The mRNA platform was applied to the development of variant-adapted vaccines to target SARS-CoV-2 variants as they emerged (38, 39). Extensive post-licensure real-world data attest to the safety and effectiveness of mRNA vaccines in curbing COVID-19–associated morbidity and mortality (40, 41). These data were valuable for expanding the landscape of mRNA vaccines and therapeutics beyond COVID-19; numerous mRNA vaccines have entered clinical development for respiratory syncytial virus, Zika virus, HIV, influenza, cytomegalovirus, varicella-zoster, and rabies virus (42).

## 3 Implementation of the mRNA platform: lessons from COVID-19

Several lessons from the COVID-19 pandemic can be leveraged to improve on implementation of mRNA vaccines and medicines.

### 3.1 Promoting transparent and accurate information-sharing to enhance uptake of novel treatments

Early in the course of pandemic, only 50–60% of the surveyed global population reported willingness to receive a COVID-19 vaccine (43). Concerns about long-term effects, low confidence in efficacy, unprecedented speed of development, and lack of communication from trusted providers were identified as barriers to COVID-19 vaccine uptake (43). Vaccine hesitancy was more prevalent in certain demographic groups, including younger age, Black race, Hispanic ethnicity, and lower educational attainment (4, 43–45). The degree of vaccine hesitancy among healthcare workers was concerning in some countries, as this population is regarded as a trusted source of information regarding COVID-19 (3, 43). Public uncertainty around non-pharmaceutical interventions (e.g., masking) and frequent revisions to vaccine policy recommendations further fueled mistrust in COVID-19 vaccination (46). For example, at the beginning of vaccination campaigns, the advice was that only one or two doses (depending upon the vaccine brand) would be needed, and no booster (47). Subsequent recognition of the reduced vaccine effectiveness in the context of emerging variants led to the recommendation of booster shots (48). In addition, mixing of vaccine brands, initially discouraged, was ultimately encouraged after finding this improved the immune response (49, 50).

The concerns about the short- and long-term side effects of the COVID-19 vaccine were echoed in parents of children aged 5–11 years following the authorization of COVID-19 vaccines for pediatric populations (3, 46). Despite the established benefit–risk profile of mRNA COVID-19 vaccines (51), acceptable safety profiles (52, 53), and the rarity of post-vaccination myocarditis in the general population (51, 54), there were parental concerns about reactions to the vaccine, fertility issues, and myocarditis, while confusion around vaccine booster recommendations fueled vaccine hesitancy (46). Motivators among parents that drove vaccine uptake for children included protection from COVID-19 and multifaceted impact of disruptions to schooling (e.g., children missing school or falling behind) (46).

These findings underscore the importance of disseminating transparent, consistent, and evidence-based messaging, to ensure confidence in and enhanced uptake of novel treatments.

### 3.2 Supporting sectors that emerged as trusted sources of information during the COVID-19 pandemic

#### 3.2.1 Employers

The Edelman Trust Barometer, a globally deployed online survey of the general population that included responses from ~33,000 individuals in 28 countries, revealed key shifts in public trust as the COVID-19 pandemic evolved (55–57). In May 2020, government was the institution most trusted by the public, compared with the media, non-government organizations (NGOs), and businesses, with increases in public trust of 5–24% since January 2020 in 10 of 11 countries surveyed, as determined by the Trust Barometer (55). By January 2021, trust in government had declined by an average of 8% globally; businesses emerged as the only institution trusted as both competent and ethical, with employers (76%) replacing other institutions (NGOs, 57%; government, 53%) as trusted sources of information (55). In 2022, trust in government and the media declined further, with a greater proportion of individuals perceiving these institutions as divisive (48 and 46%, respectively) rather than unifying force in society (36 and 35%); by contrast, businesses and NGOs were more frequently perceived as unifying (45 and 50%, respectively) than divisive (31 and 29%) (56).

A measurable impact of the role of employers during the COVID-19 pandemic was evidenced in a cross-sectional study of nursing and social-care employees in Austria, where employer recommendation affected the decision to vaccinate against COVID-19 in 19% of the 625 participants (58). These findings were echoed in a survey of 400 US-based companies, reporting that employer vaccine-adoption strategies centered on increasing conviction (e.g., sharing scientifically accurate resources), convenience (e.g., setting up onsite vaccination clinics), and reducing the cost (e.g., covering direct costs associated with vaccination) would encourage vaccination in the majority of employees (59). A viable strategy to enhance uptake is therefore to encourage vaccination through employers by disseminating evidence-based information and providing practical support. Notably, while employers appeared hesitant to mandate vaccination as a condition of employment (60), mandated vaccination seemed to have little impact on decision to vaccinate in unvaccinated employees, with 74.3% of participants responding they would rather lose their job than get vaccinated (58).

#### 3.2.2 Healthcare providers

The global response to the COVID-19 pandemic was primarily led by government, who took on the role of recommending and implementing control measures (61). The government response was prone to politicization and divisiveness (56, 62, 63), and, due to the speed of the pandemic, HCPs were not necessarily involved in the traditional way during COVID-19 vaccination campaigns. Trust in HCPs was, however, reported to be greater than in government agencies (63, 64), and was positively associated with COVID-19 vaccine behaviors in multiple studies (46, 63). A qualitative study from the United States found that HCPs were the most trusted sources of information on COVID-19 vaccinations among parents (46). Furthermore, trust in physicians was associated with COVID-19 vaccine uptake among adults in the USA; it was estimated that increasing this trust could induce at least 10% increase in vaccine and booster uptake (63). The HCPs, therefore, seem to be uniquely positioned to educate communities and support uptake of novel vaccines.

### 3.3 Equitable healthcare requires expansion of health campaigns and clinical trials to be more inclusive

Enhancing inclusion of minority groups in healthcare and representation of historically marginalized communities in clinical trials is vital to ensure trust in the development of new vaccines and therapeutics, and ultimately, equitable healthcare.

Barriers to access COVID-19 vaccines were highlighted by the disproportionate burden of COVID-19 disease on certain ethnic and racial minority groups, arising from deep-rooted structural, social, and healthcare inequalities (65–68). Vaccine hesitancy in the United States was more prevalent in minority groups that were disproportionately affected by the pandemic, including African Americans (41.6%) and Hispanic individuals (30.2%), as compared with the general US population (26.3%) (10, 66). Medical mistrust, lack of information on COVID-19 vaccines, and social disadvantage were among factors associated with increased vaccine hesitancy among these groups (10).

In 2021, 62% of the global population agreed with the statement that the pandemic was amplifying existing inequities worldwide (55). The well-documented disparity between high-and low-income populations on the Edelman Trust Barometer was especially notable in 2022 (62 vs. 47%) (56). Concerted efforts have been made to address some causes of inequity such as racial and ethnic disparities through targeted enrollments in clinical trials, including community outreach initiatives and careful monitoring of enrollment demographics to ensure rapid revision of recruitment strategies (67). Best practices which were built from past experience, with the participation of community and patient advocates in HIV research, were instrumental in driving positive change in the conduct of HIV trials in relation to participant recruitment, study design, and dissemination of findings (69). This highlights the importance of engaging community members in clinical research to raise the profile of novel therapies in the general public.

Targeted campaigns to increase healthcare availability for minority groups and improving diversity in clinical trials are viable strategies for building trust and ensuring equitable access to benefits of novel healthcare interventions, including mRNA vaccines and therapeutics.

## 4 GLP

The GLP is an initiative dedicated to generating insights into the key dimensions of societal preparedness as a way of building societal cohesion to better prepare society in times of crisis (18). The initiative draws upon quantitative and qualitative research to describe public perceptions and experiences of the COVID-19 pandemic in an effort to establish a foundational metric of public preparedness, a Societal Preparedness Index, for future emergencies (18).

The GLP survey (July 2023–September 2023) involved conducting interviews online, face-to-face, or via computer-assisted telephone in nationally representative samples in 70 countries. To be eligible for inclusion in the survey, respondents were required to be over the age of 18 years and a resident of the country where the survey was administered. To obtain a representative population, probability sampling was used for the face-to-face and computer-assisted telephone interviews. For online interviews, respondents from online panels were invited to participate, with quotas for age, gender, and region set to reflect the demographics of the national population.

**Figure 1:**
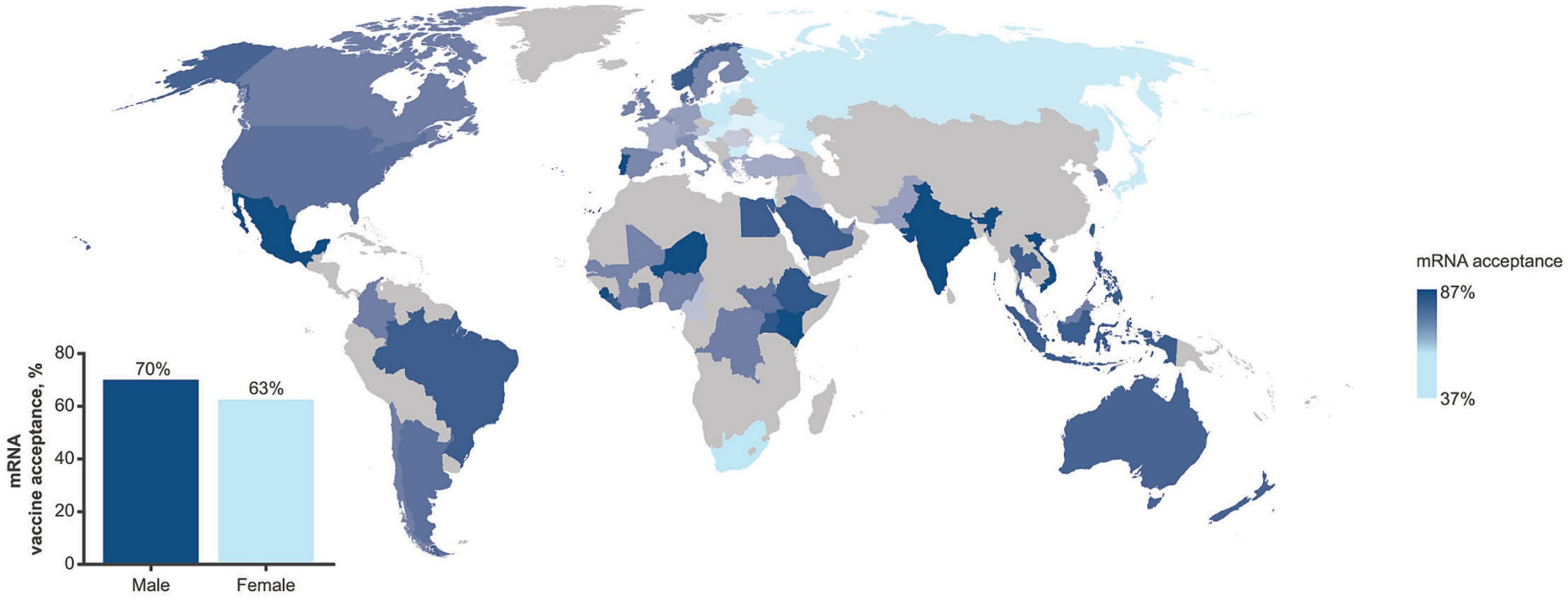
Prevalence in mRNA vaccine acceptance as assessed in the GLP survey (July 2023*–*September 2023) by geographic region. The inset shows prevalence by gender. The GLP survey involved more than 70,000 completed interviews in nationally representative samples from 70 countries.

The survey revealed stark geographic and demographic disparities in the experience of the COVID-19 pandemic and perceptions of mRNA vaccines and medicines. Among 70,781 participants who were interviewed on the mRNA vaccine acceptance, 66% affirmed that they would accept a newly approved mRNA vaccine to protect themselves; however, wide disparities were observed both by country and gender (Figure 1). In the United States and United Kingdom, the percentage of participants who were willing to accept the new mRNA vaccine was higher than average (73 and 68%, respectively). Countries where less than half of the interviewed population expressed willingness to accept the new mRNA vaccine were South Africa (37%) and central/northeastern European states (41–49%), whereas the highest level of acceptance was observed in Sierra Leone (87%). Globally, more men (70%) than women (63%) were willing to accept the new mRNA vaccine, whereas no stark age disparities were observed (18–34 years, 67%; 35–54 years: 63%; ≥55 years: 69%). Among participants who have heard of vaccines or medicines that use mRNA (*n* = 4,808), the majority agreed that mRNA vaccines were important (73%), effective (72%), and safe (68%); however, agreement was more prevalent among men (72–76%) than women (63–69%). Further, among those who reported being aware of mRNA, more than half (60%) reported that they had little knowledge about mRNA, whereas less than one-third (29%) reported they knew a lot, highlighting a discrepancy between the low prevalence of knowledge on mRNA vaccines/medicines and high prevalence of favorable perceptions on safety and efficacy of mRNA vaccines.

Challenges with acceptance of novel therapeutics are not unique to mRNA-vaccines and have been observed globally in non-emergency situations including with stem cell and gene therapy. Since its nascency, public perception of the benefits and risks of stem cell therapy has varied (70); studies have reported varying levels of trust and acceptance between countries (71) and higher levels of trust among older adults (50 years of age or above) regardless of gender (72). Similarly, attitudes toward gene therapy and gene editing also have been met with varying and complex levels of public acceptance with concerns for this therapy found to be linked to a lack of trust, education, and knowledge of risks and benefits (73–75) suggesting that continuous engagement with the public is needed to address concerns with the adoption of new medicines. The Edelman Trust Barometer Global Report for 2024 indicated sex-based differences in the acceptance of gene-based medicine, with 31% of men and 26% of women supporting gene-based medicine (76); these observations are similar to those observed with the GLP survey regarding mRNA-based vaccines. Notably, vaccine hesitancy was a challenge prior to the COVID-19 pandemic with individuals, including HCPs, choosing to delay or refuse various vaccines, possibly influenced by concerns over vaccine safety, and a lack of knowledge and motivation to get vaccinated (77, 78). Existing attitudes of vaccine hesitancy potentially influenced attitudes to COVID-19 vaccines since individuals are more likely to favor information that aligns with their existing beliefs (79). The data patterns emerging from the GLP global survey provide actionable insights to tailor strategies to increase awareness of mRNA-based vaccines and therapeutics for target populations. Among participants who were asked to report comfort with innovations in healthcare (*n* = 9,651), fewer women (33%) than men (38%) reported being comfortable with mRNA-based innovations. The attributes deemed most important for accepting a new vaccine/medicine among interviewed participants (*n* = 11,214) were proven safety (83%) and efficacy (82%), suggesting that campaigns designed to build confidence in those attributes could contribute to improving uptake. In addition, the GLP survey and related interviews revealed that the term “technology” in descriptions of mRNA-based medicines prompted negative perceptions. Public discourse and educational campaigns would therefore benefit from describing mRNA not in terms of a “technology” but as a new science-based approach to developing vaccines and therapeutics.

## 5 mRNA as a new class of medicine: application to therapeutic areas beyond infectious diseases

In addition to their application to infectious disease prevention, mRNA therapeutic approaches are being developed in oncology to induce immune-targeting responses by encoding proteins that attack and control tumors (42). Numerous mRNA therapeutic candidates against cancer are currently under investigation in clinical trials as monotherapies or combination therapies for a range of disease states, however, no mRNA-based cancer therapeutic has been approved to date (42, 80).

The capacity of mRNA to induce therapeutically relevant expression of proteins that is suitable for substituting malfunctioning or absent proteins has applications in both rare and chronic disease (33, 81). Several mRNA-based protein replacement therapies have entered phase 1 and 2 clinical trials, including LNP-encapsulated mRNA for the treatment of dysmetabolic disorders (Moderna) and cystic fibrosis (Translate Bio), and naked mRNAs for the treatment of ulcers in type 2 diabetes and heart failure (Moderna/AstraZeneca) (81). Application of mRNA vaccines in autoimmune disease is currently at the preclinical stage; however, the experimental data accrued thus far suggest that the mRNA platform is suitable for the delivery of proteins to modulate misguided immune responses in a range of autoimmune and allergic conditions (42).

Taken together, the attributes of mRNA-based products differ from other known approaches in medicine as they utilize innate biology to manufacture a broad range of preventive or therapeutic interventions, with the potential for rapid iteration. There is an urgency to apply the learnings on mRNA uptake from the pandemic and promote a broader level of confidence in this platform.

## 6 Conclusion

The cardinal feature of mRNA-based medicines is that they use intrinsic cellular mechanisms to generate proteins with therapeutic or prophylactic properties. Many decades of laboratory research in mRNA paved the way for the accelerated development of mRNA vaccines in response to the COVID-19 pandemic. However, despite favorable safety and efficacy profiles of approved mRNA COVID-19 vaccines, vaccine hesitancy was notable in the public, especially among minority and socially disadvantaged groups. As a trusted source of information, HCPs are well placed to take a greater role in building trust and discouraging the spread of misinformation. Employers are also uniquely positioned to support uptake of novel interventions during healthcare crises through transparent communication and provision of practical support to their workforce. Data from the GLP survey presented herein revealed tremendous disparities in willingness to accept new mRNA vaccines and medicines across countries, identifying women as a demographic group that should be prioritized for confidence-building strategies around mRNA vaccines and therapeutics. Concrete plans to enhance public trust and confidence in novel medicines, including the rapidly advancing field of mRNA-based therapeutics, are critical to improve clinical outcomes, reduce disease burden, and enhance the societal capacity to manage future healthcare crises.

## Data availability statement

The datasets presented in this study can be found in online repositories. The names of the repository/repositories and accession number(s) can be found at: Global Listening Project: http://www.global-listening.org.

## Ethics statement

The studies involving humans were approved by the European Society for Opinion and Marketing Research. The studies were conducted in accordance with the local legislation and institutional requirements. Written informed consent for participation was not required from the participants or the participants' legal guardians/next of kin because survey respondents had the opportunity to opt in/out of the survey and to exit the survey whenever they needed to. Respondents were also given the option to refuse to answer or state that they did not know an answer for all questions.

## Author contributions

SMI: Writing – original draft, Writing – review & editing, Conceptualization, Data curation, Formal analysis. AMR: Writing – original draft, Writing – review & editing, Conceptualization, Data curation, Formal analysis. DE: Writing – original draft, Writing – review & editing, Conceptualization, Data curation, Formal analysis. AB: Writing – original draft, Writing – review & editing, Data curation, Formal analysis, Conceptualization. HJL: Writing – original draft, Writing – review & editing, Data curation, Formal analysis, Conceptualization. MS: Writing – original draft, Writing – review & editing, Conceptualization, Data curation, Formal analysis. MR: Writing – original draft, Writing – review & editing, Conceptualization, Data curation, Formal analysis. AC: Writing – original draft, Writing – review & editing, Conceptualization. FC: Writing – original draft, Writing – review & editing, Conceptualization, Data curation, Formal analysis.

## References

[1] Wernette R Bhatnagar B Bazant E. Defining health campaigns and health campaign effectiveness. Decatur, GA: The Task Force for Global Health (2020).

[2] Ajana B Engstler E Ismail A Kousta M. Perceptions and attitudes towards COVID-19 vaccines: narratives from members of the UK public. Z Gesundh Wiss. (2022) 31:1699–715. doi: 10.1007/s10389-022-01728-w, PMID: 35789880 PMC9244123

[3] Iftekhar EN Priesemann V Balling R Bauer S Beutels P Vladez AC. A look into the future of the COVID-19 pandemic in Europe: an expert consultation. Lancet Region Health Eur. (2021) 8:100185. doi: 10.1016/j.lanepe.2021.100185 PMC8321710 34345876

[4] Fischer R Karl JA. Predicting behavioral intentions to prevent or mitigate COVID-19: a cross-cultural meta-analysis of attitudes, norms, and perceived behavioral control effects. Soc Psychol Personal Sci. (2022) 13:264–76. doi: 10.1177/19485506211019844

[5] Galadima AN Zulkefli NAM Said SM Ahmad N. Factors influencing childhood immunisation uptake in Africa: a systematic review. BMC Public Health. (2021) 21:1475. doi: 10.1186/s12889-021-11466-5, PMID: 34320942 PMC8320032

[6] Gallagher KE Kadokura E Eckert LO Miyake S Mounier-Jack S Aldea M. Factors influencing completion of multi-dose vaccine schedules in adolescents: a systematic review. BMC Public Health. (2016) 16:172. doi: 10.1186/s12889-016-2845-z, PMID: 26895838 PMC4759915

[7] Kilich E Dada S Francis MR Tazare J Chico RM Paterson P. Factors that influence vaccination decision-making among pregnant women: a systematic review and meta-analysis. PLoS One. (2020) 15:e0234827. doi: 10.1371/journal.pone.0234827 32645112 PMC7347125

[8] Roy DN Biswas M Islam E Azam MS. Potential factors influencing COVID-19 vaccine acceptance and hesitancy: a systematic review. PLoS One. (2022) 17:e0265496. doi: 10.1371/journal.pone.0265496, PMID: 35320309 PMC8942251

[9] Smith LE Amlot R Weinman J Yiend J Rubin GJ. A systematic review of factors affecting vaccine uptake in young children. Vaccine. (2017) 35:6059–69. doi: 10.1016/j.vaccine.2017.09.046, PMID: 28974409

[10] Khubchandani J Macias Y. COVID-19 vaccination hesitancy in Hispanics and African-Americans: a review and recommendations for practice. Brain Behav Immun Health. (2021) 15:100277. doi: 10.1016/j.bbih.2021.100277, PMID: 34036287 PMC8137342

[11] Shearn C Krockow EM. Reasons for COVID-19 vaccine hesitancy in ethnic minority groups: a systematic review and thematic synthesis of initial attitudes in qualitative research. SSM Qual Res Health. (2023) 3:100210. doi: 10.1016/j.ssmqr.2022.100210, PMID: 36573229 PMC9771578

[12] Alemi F Lee KH. Impact of political leaning on COVID-19 vaccine hesitancy: a network-based multiple mediation analysis. Cureus. (2023) 15:e43232. doi: 10.7759/cureus.43232 37692573 PMC10491458

[13] Quantin C Tubert-Bitter P. COVID-19 and social inequalities: a complex and dynamic interaction. Lancet Public Health. (2022) 7:e204–5. doi: 10.1016/s2468-2667(22)00033-0, PMID: 35176245 PMC8843329

[14] The Global Listening Project. (2024). Societal preparedness insights. Available at: https://global-listening.org/societal-preparedness-insights/ (accessed March 21, 2024).

[15] Chaudhary N Weissman D Whitehead KA. mRNA vaccines for infectious diseases: principles, delivery and clinical translation. Nat Rev Drug Discov. (2021) 20:817–38. doi: 10.1038/s41573-021-00283-5, PMID: 34433919 PMC8386155

[16] Parhiz H Atochina-Vasserman EN Weissman D. mRNA-based therapeutics: looking beyond COVID-19 vaccines. Lancet. (2024) 403:1192–204. doi: 10.1016/s0140-6736(23)02444-3 38461842

[17] National Institutes of Health (NIH). (2023). Decades in the making: mRNA COVID-19 vaccines. Available at: https://covid19.nih.gov/nih-strategic-response-covid-19/decades-making-mrna-covid-19-vaccines (Accessed February 22, 2024).

[18] The Global Listening Project. (2024). Home page 2024. Available at: https://global-listening.org/ (Accessed February 1, 2024).

[19] Edwards DK Carfi A. Messenger ribonucleic acid vaccines against infectious diseases: current concepts and future prospects. Curr Opin Immunol. (2022) 77:102214. doi: 10.1016/j.coi.2022.102214, PMID: 35671599 PMC9612403

[20] Nance KD Meier JL. Modifications in an emergency: the role of N1-methylpseudouridine in COVID-19 vaccines. ACS Cent Sci. (2021) 7:748–56. doi: 10.1021/acscentsci.1c00197, PMID: 34075344 PMC8043204

[21] National Human Genome Research Institute (NIH). (2024). Messenger RNA (mRNA). Available at: https://www.genome.gov/genetics-glossary/messenger-rna. (Accessed February 22, 2024).

[22] Pardi N Hogan MJ Porter FW Weissman D. mRNA vaccines—a new era in vaccinology. Nat Rev Drug Discov. (2018) 17:261–79. doi: 10.1038/nrd.2017.243, PMID: 29326426 PMC5906799

[23] Matsumura T Takano T Takahashi Y. Immune responses related to the immunogenicity and reactogenicity of COVID-19 mRNA vaccines. Int Immunol. (2023) 35:213–20. doi: 10.1093/intimm/dxac064 36566501

[24] Park JW Lagniton PNP Liu Y Xu RH. mRNA vaccines for COVID-19: what, why and how. Int J Biol Sci. (2021) 17:1446–60. doi: 10.7150/ijbs.59233, PMID: 33907508 PMC8071766

[25] John S Yuzhakov O Woods A Deterling J Hassett K Shaw CA. Multi-antigenic human cytomegalovirus mRNA vaccines that elicit potent humoral and cell-mediated immunity. Vaccine. (2018) 36:1689–99. doi: 10.1016/j.vaccine.2018.01.029, PMID: 29456015

[26] Rijkers GT Weterings N Obregon-Henao A Lepolder M Dutt TS van Overveld FJ. Antigen presentation of mRNA-based and virus-vectored SARS-CoV-2 vaccines. Vaccine. (2021) 9:848. doi: 10.3390/vaccines9080848, PMID: 34451973 PMC8402319

[27] Ci L Hard M Zhang H Gandham S Hua S Wickwire J. Biodistribution of lipid 5, mRNA, and its translated protein following intravenous administration of mRNA-encapsulated lipid nanoparticles in rats. Drug Metab Dispos. (2023) 51:813–23. doi: 10.1124/dmd.122.000980, PMID: 37208184

[28] Gebre MS Brito LA Tostanoski LH Edwards DK Carfi A Barouch DH. Novel approaches for vaccine development. Cell. (2021) 184:1589–603. doi: 10.1016/j.cell.2021.02.030, PMID: 33740454 PMC8049514

[29] Sharova LV Sharov AA Nedorezov T Piao Y Shaik N Ko MS. Database for mRNA half-life of 19 977 genes obtained by DNA microarray analysis of pluripotent and differentiating mouse embryonic stem cells. DNA Res. (2009) 16:45–58. doi: 10.1093/dnares/dsn030, PMID: 19001483 PMC2644350

[30] Cheng F Wang Y Bai Y Liang Z Mao Q Liu D. Research advances on the stability of mRNA vaccines. Viruses. (2023) 15:668. doi: 10.3390/v15030668, PMID: 36992377 PMC10051489

[31] Burdette D Ci L Shilliday B Slauter R Auerbach A Kenney M. Systemic exposure, metabolism, and elimination of [(14)C]-labeled amino lipid, lipid 5, after a single administration of mRNA encapsulating lipid nanoparticles to Sprague-Dawley rats. Drug Metab Dispos. (2023) 51:804–12. doi: 10.1124/dmd.122.001194, PMID: 37208185

[32] Urdaneta V Esposito DB Dharia P Moraga MS Anteyi K Oduyebo-Omotosho T. Global safety assessment of adverse events of special interest following 2 years of use and 772 million administered doses of mRNA-1273. Open forum. Infect Dis. (2024) 11:ofae067. doi: 10.1093/ofid/ofae067, PMID: 38500575 PMC10946654

[33] Sahin U Karikó K Türeci Ö. mRNA-based therapeutics–developing a new class of drugs. Nat Rev Drug Discov. (2014) 13:759–80. doi: 10.1038/nrd4278 25233993

[34] Karikó K. Modified uridines are the key to a successful message. Nat Rev Immunol. (2021) 21:619. doi: 10.1038/s41577-021-00608-w, PMID: 34580453

[35] The Nobel Prize. (2023). The Nobel prize in physiology or medicine 2023 press release. Available at: https://www.nobelprize.org/prizes/medicine/2023/press-release/

[36] Baden LR Essink B Kotloff K Frey S Novak R. Efficacy and safety of the mRNA-1273 SARS-CoV-2 vaccine. N Engl J Med. (2021) 384:403–16. doi: 10.1056/NEJMoa2035389, PMID: 33378609 PMC7787219

[37] Polack FP Thomas SJ Kitchin N Absalon J Gurtman A Lockhart S. Safety and efficacy of the BNT162b2 mRNA Covid-19 vaccine. N Engl J Med. (2020) 383:2603–15. doi: 10.1056/NEJMoa2034577, PMID: 33301246 PMC7745181

[38] Echaide M Chocarro de Erauso L Bocanegra A Blanco E Kochan G Escors D. mRNA vaccines against SARS-CoV-2: advantages and caveats. Int J Mol Sci. (2023) 24:5944. doi: 10.3390/ijms24065944, PMID: 36983017 PMC10051235

[39] Mir S Mir M. The mRNA vaccine, a swift warhead against a moving infectious disease target. Expert Rev Vaccines. (2024) 23:336–48. doi: 10.1080/14760584.2024.2320327, PMID: 38369742

[40] Xu W Ren W Wu T Wang Q Luo M Yi Y. Real-world safety of COVID-19 mRNA vaccines: a systematic review and meta-analysis. Vaccine. (2023) 11. doi: 10.3390/vaccines11061118, PMID: 37376508 PMC10301865

[41] Zheng C Shao W Chen X Zhang B Wang G Zhang W. Real-world effectiveness of COVID-19 vaccines: a literature review and meta-analysis. Int J Infect Dis. (2022) 114:252–60. doi: 10.1016/j.ijid.2021.11.009, PMID: 34800687 PMC8595975

[42] Zhang G Tang T Chen Y Huang X Liang T. mRNA vaccines in disease prevention and treatment. Signal Transduct Target Ther. (2023) 8:365. doi: 10.1038/s41392-023-01579-1, PMID: 37726283 PMC10509165

[43] Razai MS Chaudhry UAR Doerholt K Bauld L Majeed A. Covid-19 vaccination hesitancy. BMJ. (2021) 373:n1138. doi: 10.1136/bmj.n1138 34016653

[44] Al-Obaydi S Hennrikus E Mohammad N Lehman EB Thakur A Al-Shaikhly T. Hesitancy and reactogenicity to mRNA-based COVID-19 vaccines-early experience with vaccine rollout in a multi-site healthcare system. PLoS One. (2022) 17:e0272691. doi: 10.1371/journal.pone.0272691, PMID: 35930586 PMC9355214

[45] Coustasse A Kimble C Maxik K. COVID-19 and vaccine hesitancy: a challenge the United States must overcome. J Ambul Care Manage. (2021) 44:71–5. doi: 10.1097/JAC.0000000000000360 33165121

[46] Goulding M Ryan GW Minkah P Borg A Gonzalez M Medina N. Parental perceptions of the COVID-19 vaccine for 5-to 11-year-old children: focus group findings from Worcester Massachusetts. Hum Vaccin Immunother. (2022) 18:2120721. doi: 10.1080/21645515.2022.2120721, PMID: 36084287 PMC9746412

[47] Bert F Scaioli G Vola L Accortanzo D Lo Moro G Siliquini R. Booster doses of anti COVID-19 vaccines: an overview of implementation policies among OECD and EU countries. Int J Environ Res Public Health. (2022) 19. doi: 10.3390/ijerph19127233, PMID: 35742479 PMC9222878

[48] World Health Organization. WHO SAGE roadmap for prioritizing uses of COVID-19 vaccines. (2023). Available at: https://www.who.int/publications/i/item/WHO-2019-nCoV-Vaccines-SAGE-Prioritization-2023.1 (accessed November 10, 2023).

[49] Mahase E. Covid-19: vaccine brands can be mixed in “extremely rare occasions,” says Public Health England. BMJ. (2021) 372:n12. doi: 10.1136/bmj.n12, PMID: 33397685

[50] Capurro G Tustin J Jardine CG Driedger SM. When good messages go wrong: perspectives on COVID-19 vaccines and vaccine communication from generally vaccine accepting individuals in Canada. Hum Vaccin Immunother. (2022) 18:2145822. doi: 10.1080/21645515.2022.2145822, PMID: 36452995 PMC9762838

[51] Klamer TA Linschoten M Asselbergs FW. The benefit of vaccination against COVID-19 outweighs the potential risk of myocarditis and pericarditis. Neth Heart J. (2022) 30:190–7. doi: 10.1007/s12471-022-01677-9, PMID: 35266090 PMC8906525

[52] Anderson EJ Creech CB Berthaud V Piramzadian A Johnson KA Zervos M. Evaluation of mRNA-1273 vaccine in children 6 months to 5 years of age. N Engl J Med. (2022) 387:1673–87. doi: 10.1056/NEJMoa2209367, PMID: 36260859 PMC9634866

[53] Creech CB Anderson E Berthaud V Yildirim I Atz AM Melendez Baez I. Evaluation of mRNA-1273 Covid-19 vaccine in children 6 to 11 years of age. N Engl J Med. (2022) 386:2011–23. doi: 10.1056/NEJMoa2203315, PMID: 35544369 PMC9127699

[54] Marschner CA Shaw KE Tijmes FS Fronza M Khullar S Seidman MA. Myocarditis following COVID-19 vaccination. Heart Fail Clin. (2023) 19:251–64. doi: 10.1016/j.hfc.2022.08.012, PMID: 36863817 PMC9973554

[55] Edelman Trust Barometer. (2021). Available at: https://www.edelman.com/trust/2021-trust-barometer (Accessed April 18, 2024).

[56] Edelman Trust Barometer. (2022). Available at: https://www.edelman.com/trust/2022-trust-barometer (Accessed April 18, 2024).

[57] About the Edelman Trust Barometer. (2024). Available at: https://www.edelman.com/trust/our-methodology (Accessed April 18, 2024).

[58] Ruf AK Volkl-Kernstock S Eitenberger M Gabriel M Klager E Kletecka-Pulker M. Employer impact on COVID-19 vaccine uptake among nursing and social care employees in Austria. Front Public Health. (2022) 10:1023914. doi: 10.3389/fpubh.2022.1023914, PMID: 36438259 PMC9686277

[59] Azimi T Heller H Latkovic T Sabow A. (2021). Pharmaceuticals and medical products practice. getting to work: employers’ role in COVID-19 vaccination. Available at: https://www.trsa.org/wp-content/uploads/2021/08/McKinsey-Employers-role-in-covid-19-vaccination_final.pdf (Accessed April 18, 2024)

[60] Berkman BE Miner SA Wendler DS Grady C. The ethics of encouraging employees to get the COVID-19 vaccination. J Public Health Policy. (2022) 43:311–9. doi: 10.1057/s41271-022-00347-9, PMID: 35354922 PMC8966597

[61] Tabari P Amini M Moghadami M Moosavi M. International public health responses to COVID-19 outbreak: a rapid review. Iran J Med Sci. (2020) 45:157–69. doi: 10.30476/ijms.2020.85810.1537, PMID: 32546882 PMC7253494

[62] Gelfand M Li R Stamkou E Pieper D Denison E Fernandez J. Persuading republicans and democrats to comply with mask wearing: an intervention tournament. J Exp Soc Psychol. (2022) 101:104299. doi: 10.1016/j.jesp.2022.104299, PMID: 35469190 PMC9021555

[63] Silver D Kim Y Piltch-Loeb R Abramson D. One year later: what role did trust in public officials and the medical profession play in decisions to get a booster and to overcome vaccine hesitancy? Prev Med Rep. (2024) 38:102626. doi: 10.1016/j.pmedr.2024.102626, PMID: 38375180 PMC10874870

[64] Edelman Trust Barometer. Special report: Trust and health. (2023). Available at: https://www.edelman.com/trust/2023/trust-barometer/special-report-health(Accessed April 18, 2024).

[65] Badalov E Blackler L Scharf AE Matsoukas K Chawla S Voigt LP. COVID-19 double jeopardy: the overwhelming impact of the social determinants of health. Int J Equity Health. (2022) 21:76. doi: 10.1186/s12939-022-01629-0, PMID: 35610645 PMC9129892

[66] Evans MK. Covid's color line - infectious disease, inequity, and racial justice. N Engl J Med. (2020) 383:408–10. doi: 10.1056/NEJMp2019445, PMID: 32726526

[67] Hill J Montross D Ivarsson M. Diversity and inclusion in clinical trials: evolution throughout the development of an mRNA COVID-19 vaccine. Front Public Health. (2023) 11:1113003. doi: 10.3389/fpubh.2023.1113003 37181705 PMC10169614

[68] Tai DBG Shah A Doubeni CA Sia IG Wieland ML. The disproportionate impact of COVID-19 on racial and ethnic minorities in the United States. Clin Infect Dis. (2021) 72:703–6. doi: 10.1093/cid/ciaa815, PMID: 32562416 PMC7337626

[69] Karris MY Dubé K Moore AA. What lessons it might teach us? Community engagement in HIV research. Curr Opin HIV AIDS. (2020) 15:142–9. doi: 10.1097/coh.0000000000000605, PMID: 31895141 PMC7374765

[70] Downey R Geransar R. Stem cell research, publics' and stakeholder views. Health L Rev. (2008) 16:2

[71] Shineha R Inoue Y Yashiro Y. A comparative analysis of attitudes toward stem cell research and regenerative medicine between six countries – a pilot study. Regener Ther. (2022) 20:187–93. doi: 10.1016/j.reth.2022.04.007, PMID: 35620641 PMC9114515

[72] Aboalola D Ramadan M Baadhaim M Alsiary R Badraiq H Alghamdi T. Public awareness and understanding of stem cell treatments available in Saudi Arabia and their trust in hospitals and research centers involved in stem cell research—a cross sectional study. Front Public Health. (2024) 12:1364809. doi: 10.3389/fpubh.2024.1364809, PMID: 38628851 PMC11018913

[73] Delhove J Osenk I Prichard I Donnelley M. Public acceptability of gene therapy and gene editing for human use: a systematic review. Hum Gene Ther. (2020) 31:20–46. doi: 10.1089/hum.2019.197, PMID: 31802714

[74] McFadden BR Rumble JN Stofer KA Folta KM. U.S. public opinion about the safety of gene editing in the agriculture and medical fields and the amount of evidence needed to improve opinions. Front Bioeng Biotechnol. (2024) 12:1340398. doi: 10.3389/fbioe.2024.1340398, PMID: 38433825 PMC10904643

[75] Li Y Zhang X Xiang Z Chen T Hu Z Yang K. Public attitudes about the use of gene therapy in mainland China. JAMA Netw Open. (2023) 6:e2328352–2. doi: 10.1001/jamanetworkopen.2023.28352, PMID: 37566417 PMC10422191

[76] 2024 Edelman Trust Barometer Global Report. (2024). Available at: https://edelman.com/trust/2024/trust-barometer (Accessed April 18, 2024).

[77] Paterson P Meurice F Stanberry LR Glismann S Rosenthal SL Larson HJ. Vaccine hesitancy and healthcare providers. Vaccine. (2016) 34:6700–6. doi: 10.1016/j.vaccine.2016.10.042 27810314

[78] Larson HJ Jarrett C Eckersberger E Smith DM Paterson P. Understanding vaccine hesitancy around vaccines and vaccination from a global perspective: a systematic review of published literature, 2007–2012. Vaccine. 32:2150–9. doi: 10.1016/j.vaccine.2014.01.081 24598724

[79] Lazarus JV White TM Wyka K Ratzan SC Rabin K Larson HJ. Influence of COVID-19 on trust in routine immunization, health information sources and pandemic preparedness in 23 countries in. Nat Med. (2023) 30:1559–63. doi: 10.1038/s41591-024-02939-2 PMC11186753 38684861

[80] Lorentzen CL Haanen JB Met Ö Svane IM. Clinical advances and ongoing trials on mRNA vaccines for cancer treatment. Lancet Oncol. (2022) 23:e450–8. doi: 10.1016/s1470-2045(22)00372-2, PMID: 36174631 PMC9512276

[81] Vavilis T Stamoula E Ainatzoglou A Sachinidis A Lamprinou M Dardalas I. mRNA in the context of protein replacement therapy. Pharmaceutics. (2023) 15:166. doi: 10.3390/pharmaceutics15010166, PMID: 36678793 PMC9866414

[82] Jain S Venkataraman A Wechsler ME Peppas NA. Messenger RNA-based vaccines: past, present, and future directions in the context of the COVID-19 pandemic. Adv Drug Deliv Rev. (2021) 179:114000. doi: 10.1016/j.addr.2021.114000, PMID: 34637846 PMC8502079

[83] Kozak M Hu J. The integrated consideration of vaccine platforms, adjuvants, and delivery routes for successful vaccine development. Vaccine. (2023) 11:695. doi: 10.3390/vaccines11030695, PMID: 36992279 PMC10055765

[84] Pollard AJ Bijker EM. A guide to vaccinology: from basic principles to new developments. Nat Rev Immunol. (2021) 21:83–100. doi: 10.1038/s41577-020-00479-7, PMID: 33353987 PMC7754704

[85] Maslow JN Kwon I Kudchodkar SB Kane D Tadesse A Lee H. DNA vaccines for epidemic preparedness: SARS-CoV-2 and beyond. Vaccines. (2023) 11:1016. doi: 10.3390/vaccines11061016, PMID: 37376404 PMC10302025

[86] Lamprinou M Sachinidis A Stamoula E Vavilis T Papazisis G. COVID-19 vaccines adverse events: potential molecular mechanisms. Immunol Res. (2023) 71:356–72. doi: 10.1007/s12026-023-09357-5, PMID: 36607502 PMC9821369

